# Mercury Poisoning and the Wider Implications

**DOI:** 10.1155/carm/6656955

**Published:** 2025-12-25

**Authors:** Ravi Mani, John Dereix, Penelope Beddoes, Tom Lawton

**Affiliations:** ^1^ Intensive Care, Bradford Royal Infirmary, Yorkshire, Bradford, UK, nhs.uk

**Keywords:** critical care admissions, mercury, personal protective equipment, poisoning

## Abstract

Poisoning from mercury has the potential to affect multiple organ systems and can be fatal in some circumstances. We present a case of a patient who had both ingested and inhaled elemental mercury resulting in deposits in his pulmonary and gastrointestinal systems. The patient was admitted to the intensive care unit (ICU) as a precautionary measure but did not require any organ support. The patient was treated with chelation therapy guided by the UK National Poisons Information Service (NPIS) and was discharged from ICU after having a prophylactic appendicectomy due to mercury deposition. This case presented several challenges logistically as there was a need to follow expert guidance with regards to personal protective equipment (PPE) and waste disposal, alongside the clinical requirements.

## 1. Introduction

Mercury is a naturally occurring metal found in elemental, inorganic and organic forms. Due to its poor safety profile, mercury use is becoming less popular; however, mercury can still be found in batteries, thermometers, barometers and chemical catalysts [1]. Poisoning from mercury can have a wide range of systemic effects dependent on the type of mercury ingested quantity of mercury ingestion, route of administration and the age of exposure [1, 2]. It affects the gastrointestinal, renal and neurological systems. This is a case report of elemental acute mercury poisoning.

## 2. Case Presentation

A man in his 20s with a background of anxiety and depression was admitted to the Emergency Department (ED) after a deliberate mixed overdose of escitalopram, diazepam, isopropyl alcohol (99% ABV) and both inhalation and ingestion of around 300 mL of elemental mercury that had been heated up. The consumption of mercury was confirmed by paramedic staff who attended the scene. It had been obtained from film projectors which the patient had previously collected.

On presentation to ED, the patient baseline observations were oxygen saturations of 100% on room air, a blood pressure of 97/63 mmHg, heart rate of 136 beats/min and respiratory rate of 24/min. The patient was given immediate fluid resuscitation with IV crystalloid whilst specialist advice was sought. Clinical examination of the cardiovascular, respiratory and abdominal systems was normal, with the only positive finding on examination was an altered Glasgow Coma Score of 14 (E4V4 M6) and signs of erythema on the forehead due to some self‐harm.

### 2.1. Investigations

Preliminary investigations were undertaken including a venous blood gas, full blood count, renal and liver function, as well as a paracetamol and salicylate level. The blood results were grossly normal, except for an elevated white blood cell count (WBC) of 19.8 × 10^9^/L and low potassium (3.0 mmol/L, normal range: 3.5–5.2) and phosphate levels (0.66 mmol/L, normal: 0.8–1.5)

The paracetamol level was very slightly elevated at 7 mg/ml (normal range: 0–5) and the salicylate level was negative. The patient was not started on N‐acetyl cysteine as the paracetamol level was thought to be of little significance. An ECG showed a sinus tachycardia. Chest and abdominal X‐rays (AXRs) were performed, showing scattered radiopacities in both left and right lower zones of the lung, as well as the stomach and the oesophagus (Images 1 and 2, in Supporting Information). Furthermore, a CT scan of the abdomen helped further characterise the mercury deposits (Image 3, again in Supporting Information).

After discussion with the National Poisons Information Service (NPIS) within the United Kingdom, chelation therapy with dimercaptosuccinic acid (DMSA) was started at a dose of 250 mg, 10 times a day. As a precaution, the patient was moved to the intensive care unit (ICU) within a negative pressure environment. Whilst on the ICU, an arterial cannula was inserted for frequent blood gas analysis, and the patient continued on chelation therapy. Alongside the chelation therapy, the patient received input from the inpatient psychiatry services. At no point did the patient develop any organ dysfunction whilst on ICU.

Serum and urinary mercury levels were taken on admission; however, these were not available till 48 h postexposure. On admission, the serum mercury concentration was 3126 nmol/L, but after 10 days of chelation therapy, this had decreased to 753 nmol/L. After further specialist discussions, the patient had two further rounds of IV chelation therapy, each of 5 days.

Serial AXRs revealed mercury deposition in the appendix (Image 4, Supporting Information). After further discussions with the NPIS and the in‐hospital general surgical team, at first, a conservative approach was taken to aim for enteral excretion of the mercury, using patient positioning aiming to promote drainage of mercury from the appendix. This was unsuccessful, so the patient underwent an appendicectomy. Postoperatively, the patient was discharged from the ICU to the medical ward to complete chelation therapy. The patient was then subsequently discharged from hospital.

### 2.2. Outcome and Follow‐Up

Upon discharge from hospital, the patient was followed up by both medical and mental health services. The patient continued to have urinary and blood mercury levels measured, with further guidance was sought from NPIS, but no treatment was required. When the urinary mercury concentration was below 35 nmol/L, testing was stopped. After review, we contacted the NPIS, and we found that a urinary mercury concentration of 35 nmol/L is the cutoff value below which there is no association with risk to human health. This cutoff value has been defined by the German Human Biomonitoring Committee [3].

## 3. Discussion

This case posed both clinical and logistical challenges as well as clinical ones. The first logistical challenge was to the first responders (ambulance staff, paramedics and fire service personnel), who attended the emergency call to the patient’s home, believing they were attending a case of medication overdose and potential flammable gas exposure. As a result, they were not wearing appropriate personal protective equipment (PPE) and were identified as having a potential exposure risk. Following discussion with the NPIS, they were admitted to hospital for observation, baseline blood tests and mercury‐level monitoring. Unfortunately, as the first responders were not consented and lost to follow‐up, the results are not available.

The decision made by the ED clinical team to wear FFP3 masks although not indicated was continued as the patient was admitted to ICU, likely due to a lack of experience. As mercury is a volatile elemental metal, it actually would not be effectively filtered by FFP3 masks. The hospital’s ICU had a stock of Sundström respirator masks and procured several of their SR 299‐2 ABEK1‐Hg‐P3 R filters, manufactured to protect against mercury vapour. These do provide protection against mercury vapour greater than 13 mg/m^3^ for up to 50 h [4], far exceeding the recommended exposure standard for mercury of 0.025 mg/m^3^ of the National Occupational Health and Safety Commission (NOHSC) and American Conference of Governmental Industrial Hygienists (ACGIH) [5, 6]. The Canadian Centre for Occupational Health and Safety (CCOHS) states that to provide adequate respiratory protection up 1 mg/m^3^ requires a respirator either with a cartridge with mercury protection or supplied by air [6]. This may have been the most suitable respiratory protection in this case [6]. On reflection, obtaining the Sundström respirators was a useful relic from the COVID‐19 pandemic as the ability to insert different filters against different chemicals makes them versatile for a number of different clinical scenarios.

The expert advice stated that the exposure risk to staff was deemed to be higher from the patient’s faeces and urine, given the excretion of elemental mercury, rather than from exhaled mercury vapour. This is because elemental mercury in the gastrointestinal tract has a very poor absorption, and second, any mercury absorbed systemically would be excreted in the patient’s urine [7, 8]. Because of this, waste disposal was considered early in this case and advice sought from the NPIS and local health and safety teams. If the patient’s waste products were disposed of via normal methods, it would lead to significant contamination of the local waste and water processing infrastructure, environment and water table. It was recommended that a fluid‐resistant disposable plastic apron, nitrile gloves and face masks was the correct level of PPE for treatment of the patient and removal of waste. For the duration of the patient’s hospital admission, they used a commode, and their faeces was collected, double bagged, secured and stored in a designated container for collection by an environmental disposal agency. Their urine was similarly collected, mixed with vernagel sachets, a high absorbency powder, which solidified it, and stored in the same container. The rapid multidisciplinary team approach was integral in order to protect not just the patient and staff and enabled focussing on the multitude of issues which were presented by the case, outside of managing the patient’s treatment, which was initially directly managed by emergency and then intensive care teams.

Fortunately, during the admission, the patient suffered no significant physiological disturbance from the overdose. There is a higher risk of organ damage from mercury inhalation rather than mercury ingestion because enteral mercury is poorly absorbed, especially when there is no loss of integrity to the gastrointestinal tract [8]. Sandborgh‐Englund et al. demonstrated a direct correlation between inhaled mercury and blood mercury concentrations, which is why in this case we were so concerned about potential pneumonitis and the need for protection for staff particularly on the ICU [2, 9]. The decision to move the patient to the ICU may have seemed overly cautious in this case, especially no organ support was required; however, the presenting blood mercury concentration was 3126 nmol/L, which far exceeds the CDC blood mercury concentration where neurological side effects of mercury are seen clinically, which is over 500 nmol/L [3].

The decision to perform a prophylactic appendicectomy was informed by previous published case reports describing the appendix as a potential pooling site for elemental mercury leading to ongoing systemic absorption and chronic exposure. There are also reported cases of appendiceal rupture from pooled mercury leading to mercury peritonitis [10]. In several cases like this one, a prophylactic appendicectomy has been performed to remove this sequestered elemental mercury [11–13].

## Consent

Written consent was obtained for this case report.

## Conflicts of Interest

The authors declare no conflicts of interest.

## Funding

No funding was required for this journal.

## Supporting Information

The Supporting Information contains the radiographical images of the patient showing the mercury deposits in the chest and abdomen, with further images showing the mercury deposition in the patient’s appendix prior to appendicectomy.

## Supporting information


**Supporting Information** Additional supporting information can be found online in the Supporting Information section.

## Data Availability

The data that support the findings of this study are available on request from the corresponding author. The data are not publicly available due to privacy or ethical restrictions.
