# Supplementary material for: Mercury Poisoning and the Wider Implications
Source: Case Rep Med. 2025 Dec 25;2025:6656955. doi: 10.1155/carm/6656955 (PMC12740589; doi:10.1155/carm/6656955)
Supplement: Supplementary file 1 — Supporting Information Additional supporting information can be found online in the Supporting Information section. [file CARM-2025-6656955-s001.docx]

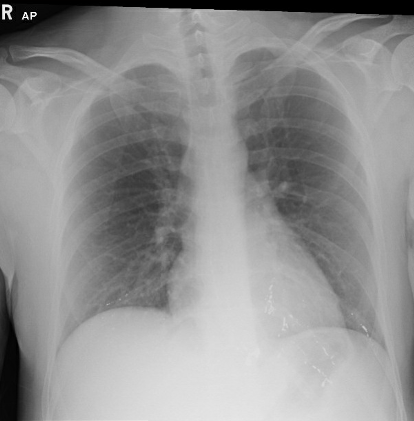


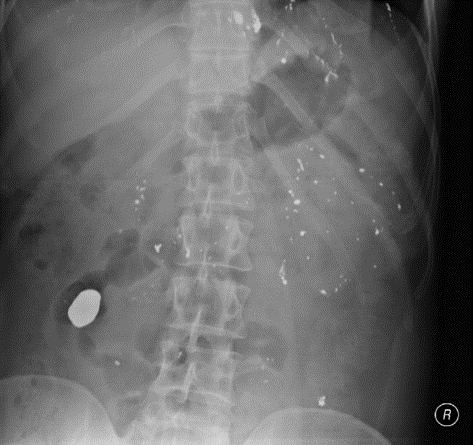


Images 1 and 2 are chest and abdominal radiographs of the patient (taken at the patient’s consent) which show mercury deposits in the chest and abdomen.


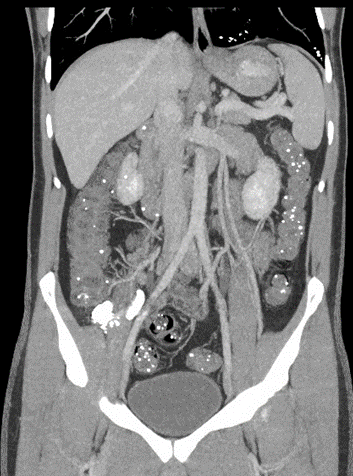


Image 3 is a coronal slice of a computerised tomography (CT) scan of the patient (taken with consent). It shows mercury deposits throughout the abdomen, with accumulation in the appendix.


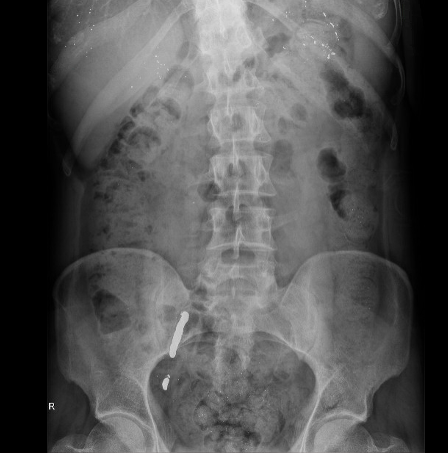


Image 4 shows mercury deposits throughout the abdomen, with accumulation in the appendix.
